# Effect of radiation-reaction on charged particle dynamics in a focused electromagnetic wave

**DOI:** 10.1038/s41598-022-23307-5

**Published:** 2022-11-10

**Authors:** Shivam Kumar Mishra, Sarveshwar Sharma, Sudip Sengupta

**Affiliations:** 1 Institute for Plasma Research, Gandhinagar, Gujarat 382428 India; 2 Homi Bhabha National Institute, Training School Complex, Mumbai, 400094 India

**Keywords:** Plasma-based accelerators, Plasma physics, Statistical physics, thermodynamics and nonlinear dynamics

## Abstract

The effect of radiation-reaction force on the dynamics of a charged particle in an intense focused light wave is investigated using the physically appealing Hartemann-Luhmann equation of motion. It is found that, irrespective of the choice of initial conditions, radiation reaction force causes the charged particle to cross the focal region, provided the particle is driven into regions where the radiation reaction force dominates over the Lorentz force, thus enhancing the forward energy gained by the particle from the intense light wave. This result is in sharp contrast to the well known result, derived in the absence of radiation reaction forces, where for certain initial conditions the particle reflects from the high intensity region of the focused light wave, thereby losing forward energy. From the perspective of energy gain, our studies clearly show that the parameter space for forward energy gain which is reduced by ponderomotive effects is compensated by radiation reaction effects. These results, which are of relevance to the present day direct laser acceleration schemes of charged particle, also agrees with that obtained using the well known Landau-Lifshitz equation of motion.

## Introduction

The relativistic motion of a charged particle placed in an electromagnetic wave is a problem of fundamental interest and is of relevance for investigating the interaction of intense radiation with matter. The problem was first solved analytically by Landau and Lifshitz^1^ for the case of a charged particle interacting with a relativistically intense, plane monochromatic electromagnetic wave, using the Hamilton-Jacobi method. The charged particle motion becomes relativistic when the cyclotron frequency of the particle in the magnetic field of the light wave ($\omega _B = e B / m c$; where *e*, *m*, *c* and *B* and are respectively the charge and mass of the charged particle, speed of light and magnetic field of the electromagnetic wave) becomes of the order (or exceeds) the frequency ($\omega$) of the wave itself i.e. $\omega _B \ge \omega$. One striking result of this study was, that a charged particle does not gain any energy from a transverse electromagnetic wave^2–4^. This may be understood from the underlying mechanism of charged particle acceleration in a transverse electromagnetic wave, which is the following. The particle which is initially at rest, first acquires a transverse velocity in the direction of the electric field vector of the light wave. If $e E / m \omega c \ge 1$, which is equivalent to $\omega _B \ge \omega$, the magnitude of the transverse velocity reaches close to the speed of light in a time which is a fraction of the wave period. At this moment the Lorentz force on the charged particle due to the magnetic field of the light wave becomes comparable to the force due to its electric field i.e. ($e ( \vec{v}_\perp /c)\times \vec{B} \sim e \vec{E}$), as a result of which the particle starts accelerating thus acquiring a relativistic velocity along the direction of propagation of the wave. This longitudinal velocity results in a large Doppler shift in the frequency seen by the particle. As a result the particle remains approximately phase locked with the wave and keeps getting accelerated till the phase of the wave, which is travelling faster than the particle, changes such that the direction of electric field reverses. The particle now starts decelerating and finally comes to a halt. This process then repeats again; as a result the charged particle does not gain any energy from the wave.

This negative result on energy gain can also be understood from the Lagrangian, $L = -\,m c^2 \sqrt{1 - v^2/c^2} + e \vec{A}. \vec{v} / c$, which represents the interaction of a charged particle with an electromagnetic wave propagating along the *z*-direction. Here $\vec{A}(t - z/c)$, which is a function of $(t - z/c)$ is the vector potential of the wave and is directed in the transverse direction. Since the transverse coordinates are cyclic, the perpendicular component of canonical momentum $\vec{{P_{\perp }}}$ is conserved. Also using $d H /dt = -\, \partial L / \partial t = c \partial L / \partial z$, we get $d H / d t = c (d p_z / dt)$, where $H = \gamma m c^2$ is the Hamiltonian and $p_z$ is the *z* component of particle momentum (The last equality is obtained by using the Lagrange’s equation of motion). This implies that $\gamma - p_z/mc = \Delta$ is a constant of motion. Using $\gamma = \sqrt{1 + p_{\perp }^2/m^2c^2 + p_z^2/m^2c^2}$, $p_z$ may be represented in terms of the constants of motion as $p_z/mc = [ 1 - \Delta ^2 + ( \vec{{P_{\perp }}} - e \vec{A}/c)^2/m^2c^2 ] / 2 \Delta$. The negative result on energy gain is intimately tied to these two constants of motion, *viz.* the perpendicular component of canonical momentum $\vec{{P_{\perp }}}$ and the parameter $\Delta$. This may be seen as follows: Consider a particle interacting with an incoming electromagnetic wave pulse. Before the pulse reaches the particle position, the particle is at rest implying $\gamma = 1$ and $\vec{A} = 0$. Therefore the constants of motion are $\Delta = 1$ and $\vec{{P_{\perp }}} = 0$. After the interaction $\vec{A} = 0$, and the final energy which is given by $\gamma = 1 + p_{z}/mc$, again turns out to be unity (as $p_z = 0$). Thus the particle does not pick up any energy from the wave.

The above discussion suggests that in order for the particle to gain energy from an electromagnetic wave, either one or both the constants of motion must be violated. It is known that a charged particle interacting with an electromagnetic wave in the presence of an external static axial magnetic field shows unbounded gain in energy when a certain resonance condition is satisfied, i.e. when the cyclotron frequency of the particle in the external magnetic field matches the Doppler shifted frequency of the wave seen by the particle. This is the well known auto-resonant particle acceleration scheme^5–7^, where one of the constants of motion viz. the perpendicular component of canonical momentum is not conserved. Another alternative way of extracting energy from a transverse electromagnetic wave is to focus the wave. This problem of charged particle motion in a focused light wave was first investigated by Feldman et al.^8^ and later in extensive detail by Kaw and Kulsrud^9^, who introduced a spatial inhomogeneity in the vector potential representing the laser field to model the effect of focusing. The dynamical equations showed that in the focused case, $\vec{{P_{\perp }}}$ remains constant whereas $\Delta = \gamma - p_{z}$ is no longer a constant of motion, its evolution being dependent on the particle position through the spatial variation of the amplitude of the vector potential. For slow spatial variation of the vector potential, using adiabatic approximation, an approximate expression for $\Delta$ showing its dependence on particle position was obtained (for calculations of $\Delta$ with higher orders of approximation, see Ref.^10^). It was found that the particle gains energy (along with forward momentum), when it is kept in the defocused region of the light wave. This region is associated with a monotonic decrease in the parameter $\Delta$. Finally in a recent work^11^, the auto-resonant scheme of charged particle acceleration has been studied in the presence of a focused light wave. The charged particle which is initially non-resonant, is accelerated by the focused pulse and brought into resonance; which is then accelerated to very high energies. It was shown that significant amount of energy gain ($\sim 45\,\mathrm{MeV}$) can be achieved at one order of magnitude lesser values of static axial magnetic field ($\sim kT$) and laser intensity ($\sim 10^{20} \,\,\mathrm{W}/\mathrm{cm}^2$). In this scheme both the constants of motion viz. the perpendicular component of canonical momentum $\vec{{P_{\perp }}}$ and the parameter $\Delta$ are violated.

Recent advances in laser facilities have resulted in renewed interest in high energy particle acceleration schemes^12,13^using optical beams produced by Chirped Pulse Amplification method^14,15^. Presently laser light can be focussed to intensities of the order of $10^{23}\,\, \mathrm{W}/\mathrm{cm}^2$^16^ (corresponding electric field $\sim 10^{12} \,\,\mathrm{V}/\mathrm{cm}$), and in near future intensities are expected to increase by two orders of magnitude or more^16^ (the corresponding electric field can reach of the order of $\sim 10^{13}\,\, \mathrm{V}/\mathrm{cm}$). In the context of particle dynamics in such high fields, it can easily be seen that the power radiated by an electron (or positron) becomes comparable to the instantaneous rate of change of energy of the particle; which in turn implies, that in this scenario, the radiation reaction force becomes comparable to the Lorentz force acting on the particle^17–19^. For a recent review of theoretical and experimental progress towards understanding of radiation reaction force, see Ref.^20^. Earlier studies on laser driven charged particle acceleration as discussed in the previous paragraph had neglected radiation reaction effects. It turns out that in the presence of radiation reaction effects both the constants of motion i.e. $\vec{P}_{\perp }$ and $\Delta$ are violated^19,21^. Recent studies^19,21–25^ have shown that, although counter-intuitive, radiation reaction forces actually lead to energy gain by the particle. Thus, at high laser intensities, the study of charged particle dynamics in a focused laser field with the inclusion of radiation reaction effects is important from the point of view of direct laser acceleration schemes^12,13^. The present article is devoted to the study of charged particle dynamics under the combined effect of focusing and radiation reaction forces. We note here that impact of radiation reaction effects on the dynamics of a charged particle in an intense ($a_0>>1$) Gaussian-beam pulse (focused light wave) has also been studied by other authors for example by Russman et al.^26^ and Harvey et al.^27^, but there exists significant differences between their work, and the work presented here. Overall dynamics of the particle is basically governed by ponderomotive effects due to focusing along with radiation reaction effects. Depending upon the choice of initial conditions, both of these effects can either lead to energy gain or energy loss. Russman et al.^26^ have studied the dynamics of a particle in a focused wave with both co-propagating and counter-propagating initial conditions. It is found that energy gain occurs for the co-propagating case whereas energy loss occurs for the counter-propagating case. Both ponderomotive effect and radiation reaction effect can lead to the same conclusion. Whereas, Harvey et al.^27^ have specifically concentrated on the interaction of a counter-propagating particle, with a Gaussian-beam pulse (head on collision) and have found that at high initial energies when radiation reaction effects become appreciable (which is also our regime of interest), the counter-propagating particle slows down and depending on the initial conditions, either passes through the focal point with reduced energy or reflects before reaching the focal point. However, it was also found by Harvey et al.^27^ that in both these cases the results are similar to that obtained with a plane wave pulse, which implies that for their choice of initial conditions, focusing (ponderomotive effect) has little impact on the dynamics. In reality, the counter-propagating particle actually slows down due to two effects viz. ponderomotive deceleration and energy loss due to radiation damping. Therefore, in order to see the two effects separately and to clearly identify the role played by radiation reaction effects, initial conditions have to be chosen judiciously; this is what we have shown in our paper.

The fundamental equation which, within the framework of classical electrodynamics, self-consistently takes into account the effect of radiation reaction forces is the covariant Lorentz-Abraham-Dirac (LAD) equation of motion^28–31^. LAD equation takes into account both the radiated energy (electromagnetic fields which are irreversibly transported to infinity) and the Schott energy which is the field energy localized at the particle and can be exchanged with its mechanical energy. However, it is well known that the LAD equation suffers from unphysical problems like pre-acceleration and runaway solutions. These unphysical problems have been widely discussed in literature^1,32–37^. Apart from the LAD equation of motion, there exists other equations of motion viz. O’Connell (FOC)^38–40^, Eliezer^41^, Landau-Lifshitz^1^, Papas^42^, Caldirola^43^, Hartemann Luhmann^44^, Yaremko^45^, Sokolov et al.^46–48^, which takes into account the radiation reaction effects and have been derived using different approaches. In our work, we have used the following well known equations for studying the effect of radiation radiation on a charged particle interacting with a focused laser pulse. One of them is the widely used Landau-Lifshitz equation of motion, which is perturbatively derived from the LAD equation, by substituting the time derivatives of four velocity in the radiation reaction term, by the Lorentz force term. The Landau-Lifshitz equation is extensively used in literature for studying the effect of radiation reaction on accelerated charged particles^19,22,25^. Although derived using a perturbative method, the Landau-Lifshitz equation does not suffer from the above mentioned unphysical problems. Besides the Landau-Lifshitz equation, there exists another physically appealing model which also does not suffer from unphysical problems; it is the Hartemann-Luhmann equation^44^. The derivation of Hartemann-Luhmann equation is based on the simple fact that the angular distribution of radiation emitted by an accelerating charged particle becomes highly asymmetric due to relativistic Doppler effect. As a result, radiation is mainly emitted in the direction of the velocity vector of the particle resulting in a net reaction force in a direction opposite to the velocity of the particle. By calculating the radiation pressure force on an accelerating charged sphere of radius “*R*” and then taking the limit $R \rightarrow 0$, final expression of radiation reaction force acting on a accelerating charged particle was obtained. An outcome of this approach is that the radiation reaction term contains the effects due to far field alone (electromagnetic fields which are irreversibly radiated away)^44,49^. The Hartemann-Luhmann equation, thus can also be derived directly from the LAD equation, by neglecting the term due to Schott energy in comparison to the term due to radiated energy in the expression for radiation reaction^21^.

**Figure 1:**
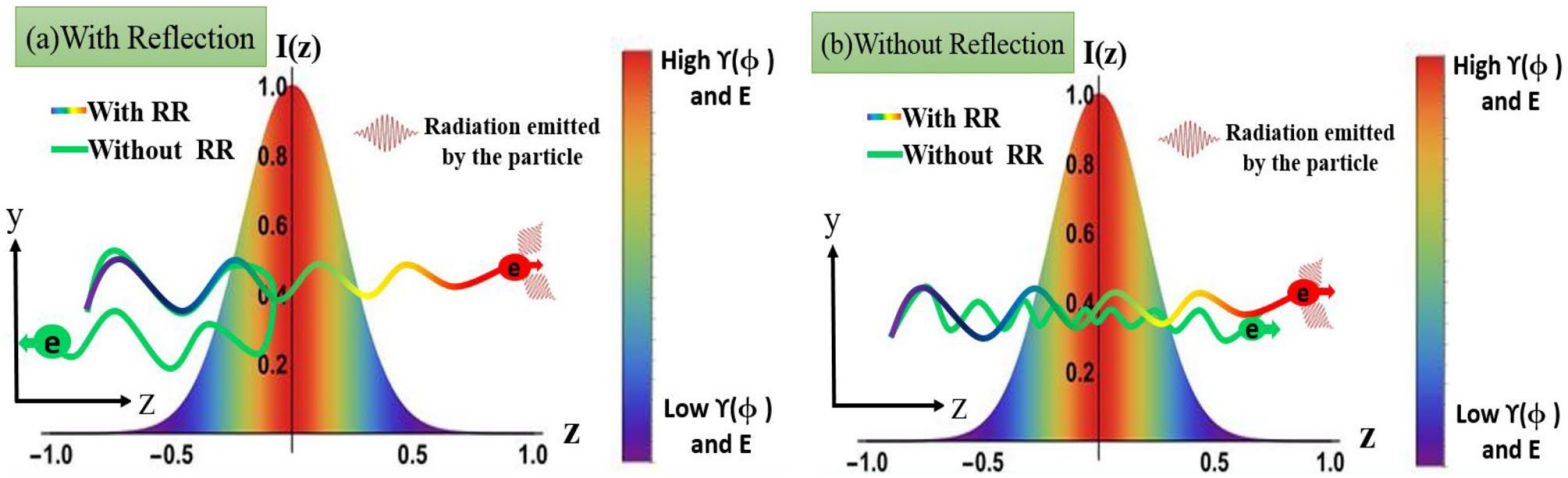
This schematic diagram compares the charged particle dynamics in a focused light wave in the absence/presence of radiation reaction (RR) effects. The variation of laser intensity (hence laser electric field)along the direction of propagation (z-axis) due to focusing is shown by a colour bar. (**a**) and (**b**)represent particle trajectories with initial conditions which respectively lead to reflection and transmission through the focal point in the absence of radiation reaction forces (the green wavy lines in (**a**) and (**b**)). It is shown that inclusion of radiation reaction forces results in transmission through the focal point in both cases, irrespective of the choice of initial conditions, provided radiation reaction force dominates over the Lorentz force. On transmission, the increase in energy of the particle is also shown by change in colour.

In the present work, we use Hartemann-Luhmann equation as well as the Landau-Lifshitz equation for studying the dynamics of a charged particle in an intense focused light wave. The Hartemann-Luhmann equation is numerically solved using a MATLAB based code used for solving implicit differential equations, whereas Landau-Lifshitz equation is solved using a MATHEMATICA based code. It is found that, independent of the choice of field configuration, with the inclusion of radiation reaction term in the equation of motion results in a monotonic decrease in the parameter $\Delta$, in the radiation reaction dominated regime. This, as mentioned above (and also shown explicitly in our earlier work^21^), is associated with energy gained by the particle from the electromagnetic wave, along with gain in forward longitudinal momentum. It is found that, irrespective of the choice of initial conditions, radiation reaction force causes the charged particle to cross the focal region, provided the particle is driven into regions where the radiation reaction force dominates over the Lorentz force, thus enhancing the forward energy gained by the particle from the intense light wave. This result is in sharp contrast to the well known result by Kaw et al.^9^, where for certain initial conditions, the particle reflects back from the high intensity regime of the focused light wave, thereby losing forward energy. The schematic diagram shown in Fig. 1 summarizes the above mentioned observations.

The organization of the paper is as follows: In Section “Governing equations” of this article, we present the basic governing equations describing the dynamics of a charged particle in a focused light wave, including radiation reaction effects. Beginning from the LAD equation, we point out the various approximations which lead to the Landau-Lifshitz and the Hartemann-Luhmann equation of motion. The Hartemann-Luhmann equation is then applied to the case of a focused electromagnetic wave. For the sake of completeness, and to get an insight into the charge particle dynamics in a focused light wave, we then discuss the reflection condition obtain by Kaw et al.^9^, without taking account of radiation reaction effects. Finally in this section, using the Hartemann-Luhmann equation and based on the equation for the parameter $\Delta$, we indicate the implications which occur due to the inclusion of radiation reaction effects. Numerical results, obtained by solving the Hartemann-Luhmann equation, describing the dynamics of a charged particle interacting with a focused wave train is presented in section “Dynamics of a charged particle in a focused light wave”. All the results obtained by solving the Hartemann-Luhmann equation of motion have been further compared with the results obtained by solving the Landau-Lifshitz equation of motion. The obtained results show that the energy gain by the particle is independent of the model equation used. Finally in section “Summary and conclusions”, we present a summary of our results.

## Governing equations

The most fundamental equation which describes the interaction of a spinless point charged particle with an electromagnetic wave including radiation reaction effects is the Lorentz-Abraham-Dirac (LAD) equation, which may be written in dimensionless covariant form as

1 $$\begin{aligned} {\dot{u}}^{\alpha } = F^{\alpha \beta } u_{\beta } + \tau _0 \left(\delta ^{\alpha }_{\beta } - u^{\alpha }u_{\beta } \right)\ddot{u}^{\beta } \end{aligned}$$

where $F^{{\alpha }{\beta }} \rightarrow e F^{{\alpha }{\beta }}/m\omega c$ is the electromagnetic field tensor, $u^{\alpha } \rightarrow u^{\alpha }/c = \gamma (1, \vec{\beta })$ is the four-velocity, $\gamma = \sqrt{1 - v^2/c^2}$, $\tau _{0} \rightarrow \omega \tau _0 =(2 / 3)(e^2 k / m c^2)$ ($\omega , \, k$ being the frequency and wave number of the electromagnetic wave) and the dot represents differentiation with respect to proper time $\tau$. We use the metric tensor $(+1,-1,-1,-1)$. The two terms in the expression for radiation reaction respectively represent the radiation reaction force due to Schott energy and the radiated energy. It is the presence of Schott term in the LAD equation which leads to unphysical problems like pre-acceleration and runaway solutions. Following Landau-Lifshitz, if the dimensionless field amplitude $a_0$ satisfies the inequality $\omega \tau _0 a_0 \ll 1$ in the instantaneous rest frame of the charged particle, then the acceleration term in the radiation reaction can be replaced by the Lorentz force term; which leads to the Landau-Lifshitz equation of motion as

2 $$\begin{aligned} {\dot{u}}^{\alpha } = F^{\alpha \beta } u_{\beta } + \tau _0 \left(\delta ^{\alpha }_{\beta } - u^{\alpha }u_{\beta } \right)\left(F^{\alpha \beta }_{, \delta }u^{\delta }u_{\gamma } + F^{\beta \gamma }F_{\gamma \delta } u^{\delta } \right)\end{aligned}$$

where $F^{\beta \gamma }_{, \delta }u^{\delta } = (d/d\tau)F^{\beta \gamma }$. Approximation of the Schott term, thus eliminates the unphysical problems associated with the LAD equation. Besides the above approximation, it can also be seen from the LAD equations that the radiation term is of order $\sim \gamma ^2$ relative to the Schott term. So in the ultra-relativistic case, where radiation reaction becomes important, Schott term may be neglected in comparison with the radiated term, thus leading to the Hartemann-Luhmann equation^44^ as (For a detailed discussion on this approximation see ref.^21^)

3 $$\begin{aligned} {\dot{u}}^{\alpha } = F^{\alpha \beta } u_{\beta } + \tau _{0} {\dot{u}}^{\beta }{\dot{u}}_{\beta } u^{\alpha } \end{aligned}$$

It is important to note that neglect of Schott term in comparison to radiation term can be rigorously justified only in the lab frame. Below, for the case of a charged particle interacting with a focused electromagnetic wave, we work with the Hartemann-Luhmann equation.

Consider the motion of a charged particle in a focused electromagnetic wave. The effect of focusing is modelled by taking the amplitude of the vector potential to be a function of space. Focusing usually means both transverse and longitudinal variation of amplitude, with amplitude increasing with decreasing distance (both radial and longitudinal distance) from the focal point. If the focal spot size is much larger compared to the Rayleigh length, the transverse variation in amplitude is much weaker compared to the longitudinal variation in amplitude^50^. It is under this approximation transverse variation may be neglected as compared to longitudinal variation in amplitude. This is the simplest way to model a focused light wave and has been used in some earlier works^9,10,26^. Following these references we have modelled the vector potential of a focused light wave as $\vec{A} = a(z) \vec{\zeta }(\phi)$, where *a*(*z*)is the spatially dependent amplitude of the vector potential, z being the direction of propagation and $\mid \vec{\zeta }(\phi)\mid = \theta (\phi)P(\phi)$ which depends only on the phase $\phi = \omega t - k z$, is product of an oscillatory part $P(\phi)\sim \cos (\phi), \, \sin (\phi)$ and a pulse shaping envelope $\theta (\phi)\sim \sin ^{2}(\phi)$ (for a wave train $\theta (\phi)$ is unity). Here *a*(*z*) is chosen in such a way that intensity of the laser light is maximum at the focal point and decreases on either side of it (as shown in Fig. 1). Transverse nature of the electromagnetic wave implies that the vector potential $\vec{A}$ (i.e. $\vec{\zeta }(\phi)$) lies in the x–y plane (plane perpendicular to the direction of propagation). The corresponding electric and magnetic field of the electromagnetic wave may be written as $\vec{E} = -\, a(z) {\vec\zeta ^{'}}(\phi)$ and $\vec{B} = -\, a(z)\{{\hat{k}} \times {\vec\zeta ^{'}}(\phi)\} + \vec{\nabla } a(z)\times \vec{\zeta }(\phi)$ respectively, where prime represents derivative with respect to $\phi$. Now the temporal and spatial components of the Hartemann-Luhmann equation of motion^21,44^ in dimensionless form can be written as

4 $$\begin{aligned} \frac{d \gamma }{dt}= & {} \vec{\beta }. \vec{E} - \tau _{0} \gamma ^{4} \left\{ \left(\frac{d \vec{\beta }}{dt}\right)^{2} + \gamma ^{2} \left( \vec{\beta }.\frac{d \vec{\beta }}{dt}\right)^{2}\right\} \end{aligned}$$

5 $$\begin{aligned} \frac{d \vec{p}}{dt}= & {} \left( \vec{E} + { \vec{\beta }} \times \vec{B}\right)- \tau _{0} \gamma ^{4} \left\{ \left(\frac{d \vec{\beta }}{dt}\right)^{2} + \gamma ^{2} \left( \vec{\beta }.\frac{d \vec{\beta }}{dt}\right)^{2}\right\} \vec{\beta } \end{aligned}$$

where the normalization used is $t \rightarrow \omega t$, $\vec{r}\rightarrow k \vec{r}$, $\vec{p} \rightarrow { \vec{p}}/{mc}$, $\vec{\beta } = \vec{v}/c$, $\vec{A} \rightarrow {e \vec{A}}/{mc^{2}}$, $\vec{E} \rightarrow {e \vec{E}}/{m \omega c}$, $\vec{B} \rightarrow {e \vec{B}}/{m \omega c}$, $\tau _0 \rightarrow \omega \tau _0$ and $dt = \gamma d \tau$. Substituting the normalized expressions for $\vec{E}$ and $\vec{B}$ in the above equations, the energy equation, and the parallel and perpendicular components of the equation of motion (parallel and perpendicular to the direction of propagation of the wave) may respectively be written as

6 $$\begin{aligned} \frac{d \gamma }{dt}= & {} -a \vec{\beta }. \vec{\zeta }' - \tau _{0} R_{h} \end{aligned}$$

7 $$\begin{aligned} \frac{d p_{z}}{dt}= & {} - a \vec{\beta }. \vec{\zeta }' + \frac{da}{dz} \vec{\beta }. \vec{\zeta } - \tau _{0} R_{h} \beta _{z} \end{aligned}$$

8 $$\begin{aligned} \frac{d \vec{p}_{\perp }}{dt}= & {} -a \left(1 - \beta _z \right) \vec{\zeta }' - \frac{da}{dz}\beta _z \vec{\zeta } - \tau _{0} R_{h} \vec{\beta }_{\perp } \end{aligned}$$

where $\beta _{z}$ is the z-component of velocity, $\gamma = \sqrt{1 + p_z^2 + p_\perp ^2}$ and $R_h = \gamma ^4({\dot{\beta }}^2 + \gamma ^2 ( \vec{\beta }.\dot{ \vec{\beta }})^2)$. Here “dot” represents derivative w.r.t to lab time. To get an insight into the dynamics of the charged particle, we first consider the case without the radiation reaction term *i.e.* $\tau _0 \rightarrow 0$. This was the case first considered by Kaw et al.^9^, which we present here for the sake of completeness. Using $(1 - \beta _z) \vec{\zeta }^{'} = d \vec{\zeta }/dt$ and $(da/dz)\beta _{z} = da/dt$, Eq. (8) may be integrated, which leads to conservation of perpendicular component of canonical momentum as

9 $$\begin{aligned} \vec{p}_{\perp } + a \vec{\zeta } = \vec{P}_{\perp 0} \end{aligned}$$

where $\vec{P}_{\perp 0}$ is a constant of motion and $\mid { \vec{\zeta }(\phi)}\mid$ is a purely oscillatory function of $\phi$ ($\sim \sin (\phi), \, \cos (\phi)$). It is thus clear from the above equation, that in order to conserve $\mid \vec{P}_{\perp 0} \mid$, the amplitude (absolute peak value) of perpendicular component of particle momentum ($\vec{p}_{\perp }$)increases as the particle moves towards the focal point (regions of increasing *a*) and decreases as it moves away from the focal point (region of decreasing *a*). Further defining $\Delta = \gamma - p_z$, the longitudinal component of momentum may be written in terms of $\vec{P}_{\perp 0}$ and $\Delta$ as

10 $$\begin{aligned} p_z = \frac{1 - \Delta ^2}{2 \Delta } + \frac{( \vec{P}_{\perp 0} - a \vec{\zeta })^2}{2 \Delta } \end{aligned}$$

The equation for $\Delta$ may be obtained by subtracting Eq. (7) from Eq. (6) as

11 $$\begin{aligned} \frac{d\Delta }{dt}= -\, \frac{da}{dz} \vec{\beta }. \vec{\zeta } - \tau _{0} R_{h} \left(1-\beta _z \right)\end{aligned}$$

It is clear from above that, in the absence of radiation reaction ($\tau _0 \rightarrow 0$) and in the unfocused case (i.e. $da/dz = 0$), $\Delta$ is a constant of motion and hence is entirely determined by the initial conditions. In such a case the knowledge of initial conditions (*i.e.* $\vec{P}_{\perp 0}$ and $\Delta$), completely specifies the perpendicular and longitudinal component of momentum of the particle. However, in the present case, $\Delta$ is no longer a constant of motion and varies with the position of the particle. Taking $\tau _0 = 0$ and assuming the amplitude of the vector potential “*a*” to be a slowly varying function of position “*z*”, the particle dynamics may approximately be divided into a slow guiding center motion and a fast motion around the guiding center^9^. To extract the slow guiding center motion, we may write $\dot{p_z} \approx \overline{\dot{p_z}}$ and ${\dot{\Delta }} \approx \overline{{\dot{\Delta }}} \approx (d \Delta / dz)\overline{p_z}$^51–53^, where the over-line represents averaging over fast variation and the “dot” represents derivative with respect to proper time ($\tau$; and $dt = \gamma d \tau$). Thus averaging over fast variation, the equation for slow temporal variation of the longitudinal momentum of the particle (from Eq. (7)), may now be written as

12 $$\begin{aligned} \frac{d {p_{z}}}{d\tau }\approx & {} - \overline{a \vec{p}. \vec{\zeta }'} + \overline{\frac{da}{dz} \vec{p}. \vec{\zeta }} \nonumber \\\approx & {} -a \overline{\vec{P}_{\perp 0}. \vec{\zeta }'} + a^2 \overline{ \vec{\zeta }. \vec{\zeta }'}+ \frac{da}{dz}\overline{ \vec{P}_{\perp 0}. \vec{\zeta }} - a \frac{da}{dz}\overline{\zeta ^{2}} \nonumber \\\approx & {} -\frac{1}{4} \frac{d a^2}{d z} \end{aligned}$$

where $\gamma \vec{\beta } = \vec{p}$ and in the second step, we have used Eq. (9) and the fact that $\vec{\zeta }$ is perpendicular to the direction of propagation of the wave (i.e. z). Taking $\vec{\zeta }(\phi)$ to be a purely sinusoidal function of $\phi$ i.e. $\theta (\phi)= 1$, we get $\overline{\zeta ^{2}} = 1/2$ and the other terms vanish. The behaviour of particle dynamics may be understood from the above averaged equation in the following way. Eq. (12) shows that the ponderomotive force ($[-1/4] da^2/dz$) pushes the particle away from the high intensity region. As the particle moves towards the focal point (region of high intensity) the average value of $p_z$ decreases monotonically with time, and for certain initial conditions may even vanish before reaching the focal point. For such cases, the particle will reflect from the high intensity region, and thus it will not gain any forward energy from the light wave. On the other hand if the particle either starts from the defocused region or from the focused region with sufficient amount of initial forward momentum so that it crosses the focal point and reaches the defocused region, it will be ponderomotively pushed away from the focal point by the light wave and hence will gain a large amount of forward momentum (hence energy) from the defocused region. This indicates that in order to extract substantial amount of forward energy, the particle must start from the defocused region. The above qualitative discussion on particle dynamics may be put on a quantitative footing by integrating the averaged equation for $\Delta$ as follows. Using ${\dot{\Delta }} \approx \overline{{\dot{\Delta }}} \approx (d \Delta / dz)\overline{p_z}$, we get

13 $$\begin{aligned} \frac{d\Delta }{d z}\approx & {} - \frac{da}{dz} \frac{\overline{ \vec{p}. \vec{\zeta }}}{\overline{p_z}} \nonumber \\\approx & {} \frac{1}{2} a \frac{da}{dz} \frac{2 \Delta }{1 - \Delta ^2 + P_{\perp 0}^2 + a^2 / 2} \end{aligned}$$

where, as before, in the second step we have used Eq. (9) and $\overline{p_z}$ is evaluated using Eq. (10) taking $\vec{\zeta }(\phi)$ to be a purely sinusoidal function of $\phi$. Multiplying both sides by $\Delta$, Eq. (13), immediately gives

14 $$\begin{aligned} \frac{d \Delta ^2}{d a^2} \approx - \frac{\Delta ^2}{\Delta ^2 - P_{\perp 0}^{2} - a^{2}/2 - 1} \end{aligned}$$

which when integrated yields $\Delta$ as a function of position *z* as,

15 $$\begin{aligned} \Delta \approx 1 + \frac{P_{\perp 0}^2}{2} + \frac{a(z_0)^2}{4} - \left[ \frac{1}{4} P_{\perp 0}^2 \left(P_{\perp 0}^2 + a(z_0)^2 \right)+ \frac{1}{16} a(z_0)^4 + \frac{1}{2} \left(a(z_0)^2 - a^2 \right)\right] ^{1/2} \end{aligned}$$

where $a(z_0)$ is the value of *a* at the initial position $z_0$ from where the particle is assumed to start from rest (i.e. $\Delta = 1$). From the above expression of $\Delta$ it is clear that, as the particle moves towards the focal point (*i.e.* increasing a), the term in the square root decreases ($\Delta$ increases) and will vanish at a particular position ($z_{ref}$) before the particle reaches the focal point, provided the following inequality is satisfied

16 $$\begin{aligned} a_{z_{ref}}^{2} = \left[ \frac{1}{2} P_{\perp 0}^2 \left(P_{\perp 0}^2 + a(z_0)^2 \right)+ \frac{1}{8} a(z_0)^4 + a(z_0)^2 \right] < a_0^2 \end{aligned}$$

where $a_0$ and $a_{z_{ref}}$ are the amplitude of the vector potential at the focal point and at the reflection point ($z_{ref}$)respectively. Beyond this position, $\Delta$ becomes complex, which physically implies that the particle cannot go into regions of $a > a_{z_{ref}}$ and will thus reflect from $z_{ref}$. Since *a*(*z*) is known, the point of reflection $z_{ref}$ may be evaluated from the value of $a_{z_{ref}}$. As stated before, the average forward longitudinal momentum decreases as the particle moves towards the focal point and vanishes at the point of reflection ($z_{ref}$). This may be seen by substituting the expression for $\Delta$ at the reflection point which is $\Delta _{ref} = 1 + P_{\perp 0}^2/2 + a(z_0)^2/4$, in the expression for average longitudinal momentum $\overline{p_z} = (1 - \Delta _{ref}^2 + P_{\perp 0}^2 + a_{z_{ref}}^2/2)/ 2 \Delta _{ref}$, which gives $\overline{p_z} = 0$. On the contrary, if the particle starts with the initial conditions, such that $a_{ref}^2 > a_0^2$, $\Delta$ will remain real during the entire motion and the particle will cross the focal point and enter the defocused region. In the defocused region, with decreasing *a*, $\Delta$ decreases and eventually becomes less than unity for $a < a(z_0)$. This results in large gain in forward longitudinal momentum as seen from Eq. (10) with concomitant gain in energy. Thus, as also stated before, the particle gains energy from the defocused region of the electromagnetic wave.

To predict, how the above dynamical behaviour of the particle changes in the presence of radiation reaction forces, we note that the radiation reaction term “$\tau _0 R_{h} (1-\beta _{z})$” in Eq. (11) is always a positive number. Therefore in the radiation reaction dominated regime, where the radiation reaction force dominates over the Lorentz force, Eq. (11) shows that the parameter $\Delta$, monotonically decreases with time. This behaviour of the parameter $\Delta$ along with the definition of $\gamma = \sqrt{1 + p_{\perp }^{2} + p_z^2}$ implies that for a particle starting from rest, increase in its forward energy is associated with the increase in its longitudinal momentum. This is in conformity with the intuitive understanding evolved for the case which is without radiation reaction effects. In the next section, we present numerical solutions to Hartemann-Luhmann and Landau-Lifshitz equation of motion using different initial conditions.

## Dynamics of a charged particle in a focused light wave

In this section, we present numerical solution to Eqs. (6)–(8), obtained using an in-house developed MATLAB based test particle code. These equations are solved for a particle interacting with a focused light wave. The vector potential corresponding to a focused wave train may be written as,

17 $$\begin{aligned} A(\phi ,z)= a \left(\delta \cos (\phi){\hat{x}} + g \sqrt{1-\delta ^{2}} \sin (\phi){\hat{y}} \right)\end{aligned}$$

where to model the effect of focusing, the functional form of amplitude *a*(*z*)is taken as,

$$\begin{aligned} a(z)= {\left\{ \begin{array}{ll} a_0 (1 + \varepsilon z),&{} \text {for } z\le 0\\ a_0 (1 - \varepsilon z), &{} \text {for } z\ge 0 \end{array}\right. } \end{aligned}$$

**Figure 2:**
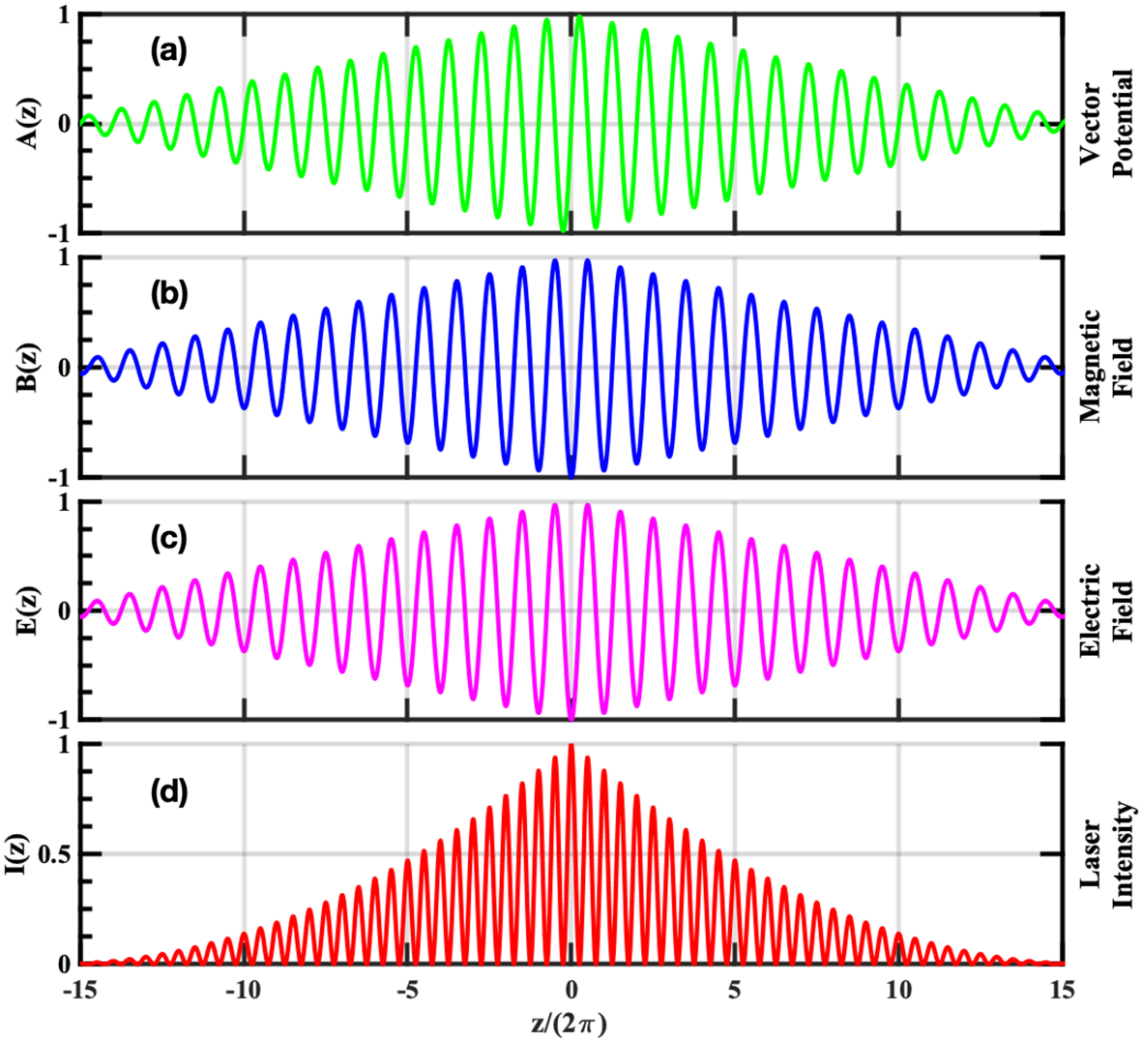
In this schematic diagram the (**a**)–(**d**), respectively represent the variation of the vector potential, magnetic field, electric field and intensity of the laser with respect to the z-coordinate. The figures show that the laser fields and laser intensity are maximum at the focal point ($z=0$) and decreases on either side of it.

Here, $\varepsilon \sim \lambda / F \ll 1$ ($\lambda$ and *F* respectively being the wavelength and the Focal length), $\mid z \mid \le 1 /\varepsilon$ and $\delta \in [0,1]$ where $\delta = 0,\,1$ and $\delta = 1/\sqrt{2}$ correspond to linear and circular polarization respectively; and $g = \pm 1$ respectively correspond to right and left handed polarization. We note here that for $\lambda \ll F$ (large number of wavelengths within the focal length) Maxwell’s equations are approximately satisfied. Further as $\varepsilon \rightarrow 0$, the discontinuity in magnetic field which is of order $\sim 2 \varepsilon$ becomes vanishingly small. For our numerical solutions we have used $\varepsilon \sim 10^{-4}$. To get a feel for the field and intensity profiles for a focused wave train, normalized laser fields and the laser intensity corresponding to the normalized vector potential given by Eq. (17)are plotted in Fig. 2, for $\delta =0$, $a_{0} = 1$ and $\varepsilon \sim 0.03$. In this schematic diagram, the sub-figures (a)–(d) respectively represent the variation of vector potential, magnetic field, electric field and intensity of the laser over $\sim 30$ cycles of the laser period. The laser field is maximum at the focal point ($z=0$) and decreases on either side of the focal point.

We now present results obtained for a charged particle interacting with a linearly polarized ($\delta = 0$), intense, focused wave train (for our numerical work, we have chosen $\varepsilon \sim 10^{-4}$). The intensity of the wave at the focal point is chosen to be $a_{0}=1000$ ($\sim 10^{24} \,\, {\rm W}/{\rm cm}^2$), and $\tau _{0} \approx 1.8 \times 10^{-8}$ (for a $1 \mu$ wavelength laser). For our numerical work, we have used three sets of initial conditions. For the first two sets, the initial conditions are chosen in such a way that the inequality given by Eq. (16) is satisfied. For these two sets, the particle is assumed to start from rest (${p}=0$) and, from initial positions $z_{0}=-\,\,9500$ and $z_0 = -\,9869$ with $a(z_0)= 50$ and $a(z_0)= 13.1$ respectively. For both these sets, the phase of the wave initially seen by the particle is chosen such that the perpendicular canonical momentum vanishes (i.e. $\vec{P}_{\perp 0} = 0$). For these initial conditions $a_{z_{ref}} \sim 885.3 < a_0$ and $a_{z_{ref}} \sim 62.7 < a_0$ respectively; thus for both these cases, as per Eq. (16) it is expected that during the motion, the parameter $\Delta$, will become complex and the particle will eventually reflect from the high intensity region. The basic difference between the above two sets is that, near the reflection point, in the first case radiation reaction force dominates over the Lorentz force *i.e.* $\tau _0 a_{z_{ref}}^3 \sim 12 > 1$, whereas in the second case it is the other way around i.e. $\tau _0 a_{z_{ref}}^3 \sim 4 \times 10^{-3}<< 1$. For the third set, the choice of initial conditions is such that the above mentioned inequality (Eq. (16)) is violated. For this set, the particle is assumed to start from rest ($\vec{p}=0$), from the initial position $z_{0}=-\,9450$, which is now placed closer to the focal point than that in the previous cases. As before, the phase of the wave initially seen by the particle is chosen in such a way that the perpendicular canonical momentum vanishes (i.e. $\vec{P}_{\perp 0} = 0$) and $a(z_0)= 55$. For these initial conditions and for the same laser and focusing parameters, $a_{z_{ref}} \sim 1070.9 > a_0$; thus as per Eq. (16), the parameter $\Delta$ will remain real throughout the motion and the particle is expected to pass through the focal point.

**Figure 3:**
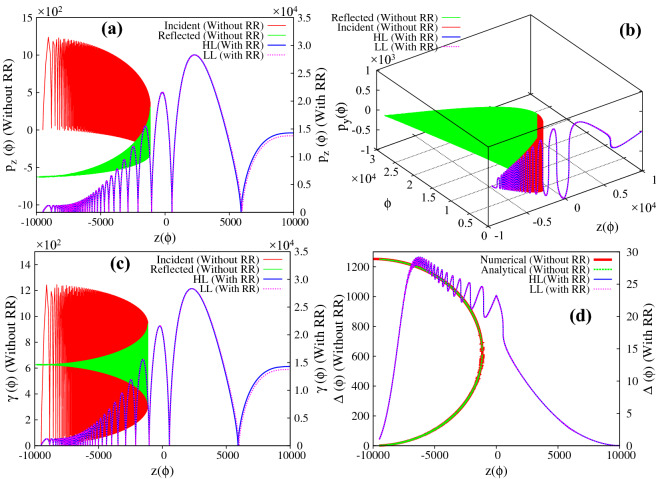
(**a**)–(**d**) Respectively represent the evolution of longitudinal and transverse momentum, the energy and the parameter $\Delta$, for a charged particle interacting with a linearly polarized ($\delta = 0$), intense, focused electromagnetic wave, in the absence/presence of radiation reaction effects. The intensity at the focal point is chosen as $a_0 = 1000$ ($\sim 10^{24}\,\, {\rm W}/{\rm cm}^2$) and $\tau _0 \approx 1.8 \times 10^{-8}$. The initial conditions are $z_{0} = -\,9500$, $\vec{p} = 0$, $P_{\perp 0} = 0$ and $a(z_0)= 50$, which satisfy the inequality given by Eq. (16) ($a_{z_{ref}} = 885.3 < a_0$; see text). The red and green curves in (**a**)–(**c**) respectively represent the forward and reflected motion of the particle in the absence of radiation reaction effects (values correspond to y-axis on the left), and the corresponding value of the parameter $\Delta$ is represented in (**d**) where the red and green curve respectively represent the numerical values and the analytical expression for $\Delta$. The blue and magenta curves in the (**a**)–(**d**) respectively represent the dynamics in the presence of radiation reaction effects (values correspond to y-axis on the right) as governed by Hartemann-Luhmann and Landau-Lifshitz equation of motion. Note that the apparent discrepancy in the initial conditions seen in the above figures for without and with radiation reaction is due to the use of different axes i.e. left y-axis is for without radiation reaction and right y-axis is for with radiation reaction effects.

**Figure 4:**
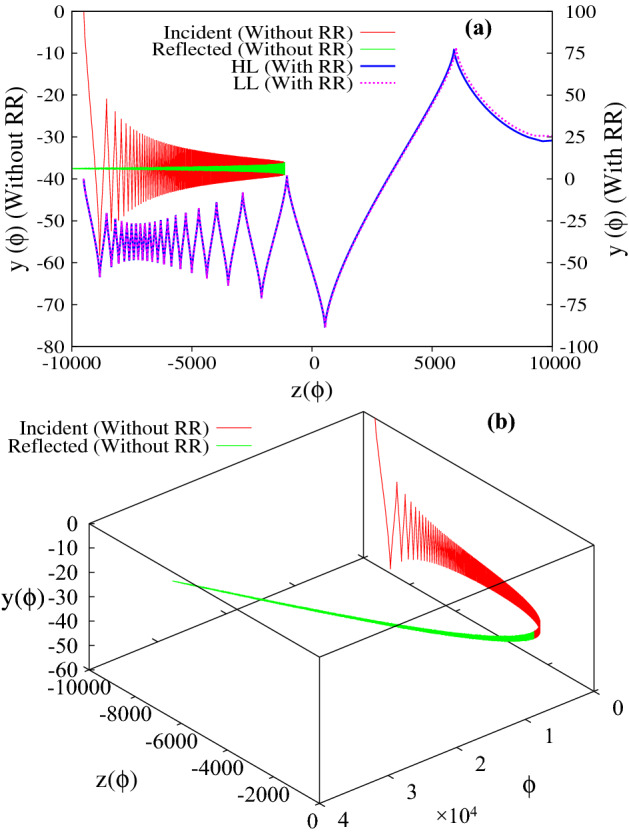
(**a**), (**b**) Represent the trajectory of the particle in the absence/presence of radiation reaction effects. The chosen initial conditions and the laser parameters are same as in Fig. 3. The red and green curves respectively represent the forward and reflected motion of the particle in the absence of radiation reaction (values correspond to y-axis on the left). The blue and magenta curve in (**a**) respectively represent the trajectory of the particle in the presence of radiation reaction effects (values correspond to y-axis on the right) as obtained by solving the Hartemann-Luhmann and the Landau-Lifshitz equation of motion. Note that the apparent discrepancy in the initial conditions seen in (**a**) for without and with radiation reaction is due to the use of different axes i.e. left y-axis is for without radiation reaction and right y-axis is for with radiation reaction effects.

**Figure 5:**
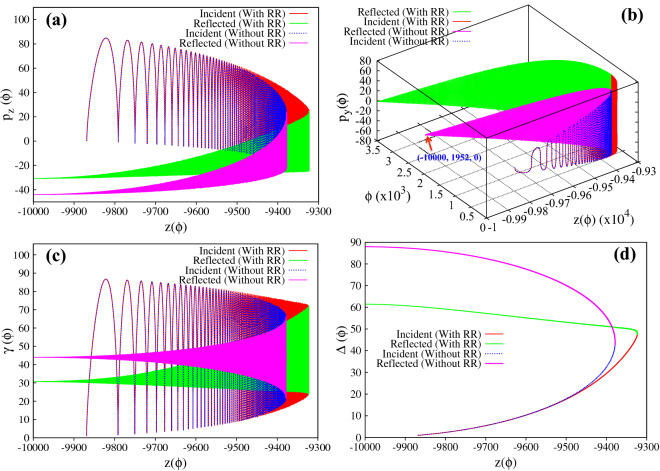
(**a**)–(**d**) Respectively represent the evolution of longitudinal and transverse momentum, the energy and the parameter $\Delta$, for a charged particle interacting with a linearly polarized ($\delta = 0$), intense, focused electromagnetic wave, in the absence/presence of radiation reaction effects. The intensity at the focal point is chosen as $a_0 = 1000$ ($\sim 10^{24}\,\, {\rm W}/{\rm cm}^2$) and $\tau _0 \approx 1.8 \times 10^{-8}$. The initial conditions are $z_{0} = -\,9869$, $\vec{p} = 0$, $P_{\perp 0} = 0$ and $a(z_0)= 13.1$, which satisfy the inequality given by Eq. (16) ($a_{z_{ref}} = 62.7 < a_0$; see text). The blue and magenta curves in (**a**)–(**d**) respectively represent the forward and reflected motion of the particle in the absence of radiation reaction effects, whereas red and green curves in the (**a**)–(**d**) respectively represent the forward and reflected motion of the charged particle in the presence of radiation reaction effects.

**Figure 6:**
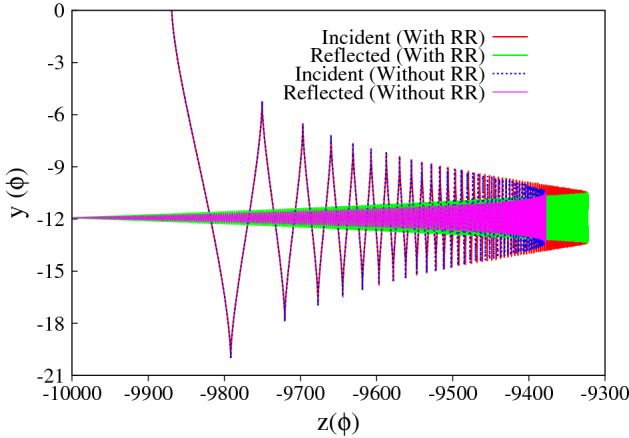
Figure represents the trajectory of the charged particle. The blue and magenta curves represent the forward and reflected motion of the particle in the absence of radiation reaction effects, whereas red and green curves respectively represent the forward and reflected motion of the charged particle in the presence of radiation reaction effects

**Figure 7:**
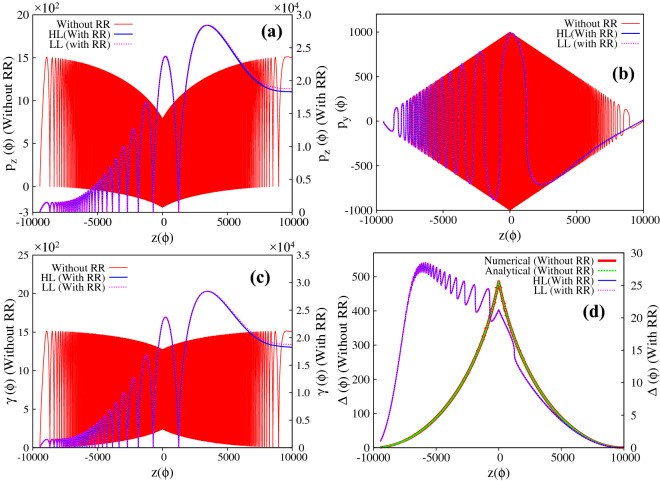
(**a**)–(**d**) Respectively represent the evolution of longitudinal and transverse momentum, the energy and the parameter $\Delta$, for a charged particle interacting with a linearly polarized ($\delta = 0$), intense, focused electromagnetic wave, in the absence/presence of radiation reaction effects. The intensity at the focal point is chosen as $a_0 = 1000$ ($\sim 10^{24}\,\, {\rm W}/{\rm cm}^2$) and $\tau _0 \approx 1.8 \times 10^{-8}$. The initial conditions are $z_{0} = -\,9450$, $\vec{p} = 0$, $P_{\perp 0} = 0$ and $a(z_0)= 55$, which violate the inequality given by Eq. (16) ($a_{z_{ref}} = 1070.9 > a_0$; see text). The red curve in (**a**)–(**c**) represent the motion of the particle in the absence of radiation reaction effects (values correspond to y-axis on the left), and the corresponding value of the parameter $\Delta$ is represented in (**d**) where the red and green curve respectively represent the numerical values and the analytical expression for $\Delta$. The blue and magenta curves in the (**a**)–(**d**) respectively represent the dynamics in the presence of radiation reaction effects (values correspond to y-axis on the right) as governed by Hartemann-Luhmann and Landau-Lifshitz equation of motion. Note that the apparent discrepancy in the initial conditions seen in (**a**) for without and with radiation reaction is due to the use of different axes i.e. left y-axis is for without radiation reaction and right y-axis is for with radiation reaction effects

**Figure 8:**
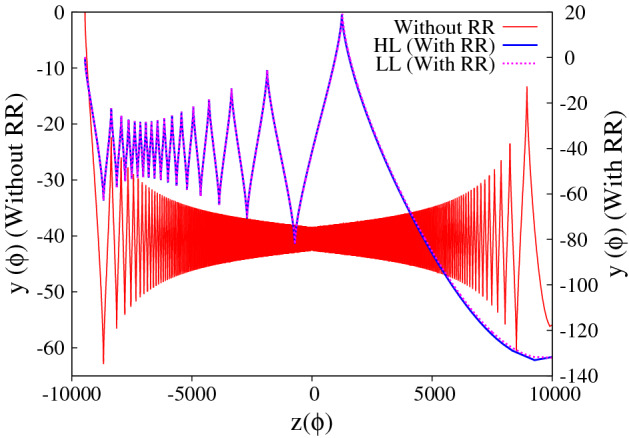
Represent the trajectory of the particle in the absence/presence of radiation reaction effects. The chosen initial conditions and the laser parameters are same as in Fig. 7. The red curve represent the motion of the particle in the absence of radiation reaction (values correspond to y-axis on the left). The blue and magenta curve respectively represent the trajectory of the particle in the presence of radiation reaction effects (values correspond to y-axis on the right)as obtained by solving the Hartemann-Luhmann and the Landau-Lifshitz equation of motion. Note that the apparent discrepancy in the initial conditions seen in the above figure for without and with radiation reaction is due to the use of different axes i.e. left y-axis is for without radiation reaction and right y-axis is for with radiation reaction effects.

For the first set of initial conditions, the numerical results are presented in Figs. 3 and 4. In Fig. 3a–d respectively represent the longitudinal ($p_z$) and transverse ($p_{y}$) component of particle momentum, the energy and the parameter $\Delta$; and Fig. 4 represents the trajectory of the particle. We first discuss the particle dynamics in the absence of radiation reaction effects. In Figs. 3a–c and 4, the red and green curves, respectively represent the forward and the reflected motion of the particle in the absence of radiation reaction effects (values correspond to y-axis on the left). The red curves in Fig. 3a and b respectively show that as the particle approaches the focal point (increasing *a*), the average longitudinal momentum decreases monotonically and approaches zero, whereas the amplitude of the transverse momentum simultaneously increases. After reflection (represented by the green curves), as the particle moves away from the focal point (decreasing *a*), the absolute value of average longitudinal momentum increases and the particle leaves the focal region with a finite value of longitudinal momentum in the opposite direction, whereas the amplitude of the transverse momentum decreases and eventually goes to zero. These numerical results are respectively in conformity with Eqs. (12) and (9). The corresponding energy is shown in Fig. 3c, which shows that for our choice of parameters the average energy which remains with the particle after reflection is around $\gamma \sim 600$ ($\sim 0.3 \,\, {\rm GeV}$). It is to be noted that the average energy does not change throughout the motion, which is in conformity with the average of Eq. (6) ($\overline{d \gamma / d \tau } \approx 0$, with $R_h = 0$). Finally, Fig. 3d shows the evolution of the parameter $\Delta$ as a function of *z* coordinate, which continuously increases throughout the motion as shown by the red curve. The green curve on top of the red curve is a plot of the analytical expression of $\Delta$ as given by Eq. (15), which clearly shows an excellent match with the numerical result. The particle trajectory is presented in Fig. 4 (red and green curves, respectively represent the forward and the reflected motion of the particle, with values corresponding to the y-axis on the left), where Fig. 4a represents the trajectory in configuration space and Fig. 4b presents its temporal evolution. The reflection of the particle at $\phi \sim 0.5 \times 10^{4}$ and $z = -\, 1.1 \times 10^{3}$ is clearly visible in Fig. 4b.

For the same set of initial conditions, in the presence of radiation reaction effects the particle dynamics which is now governed by the Hartmemann-Luhmann equation, exhibit dramatic changes. (See the blue curve in sub-figures Figs. 3a, c, d and 4a with values corresponding to y-axis on the right). Initially when the radiation reaction effects are weak, the average longitudinal momentum $\overline{p_z}$ remains almost constant (see blue curve in Fig. 3a; in fact it shows a slight decrease, which is in agreement with the behaviour governed by the Lorentz force terms in Eq. (7)), and later increases monotonically when the radiation reaction term starts dominating over the Lorentz force term. This dominance of the radiation reaction term over the Lorentz force term can also be seen from the evolution of the parameter $\Delta$ (see Fig. 3d) which shows that $\Delta$ which was initially increasing, starts monotonically decreasing when the intensity becomes sufficiently large ($a \sim 6.3 \times 10^{2}$, intensity $\sim 4 \times 10^{23}\,\, {\rm W}/{\rm cm}^2$) which happens when the particle reaches around $z \sim -0.6 \times 10^{4}$. From this location onwards, the radiation reaction term starts dominating over the Lorentz force term. Simultaneously, from around the same location *i.e.* $z \sim -0.6 \times 10^{4}$ , the average longitudinal momentum begins to increase monotonically and the particle eventually passes through the focal point with a finite amount of longitudinal momentum. The transverse momentum on the other hand shows a behaviour which is similar to the earlier case, i.e.when radiation reaction effects are absent or weak . The amplitude of the transverse momentum of the particle increases as it approaches the focal point and diminishes as it passes through the focal point, eventually becoming zero as it exits the focal region (see blue curve in Fig. 3b). Therefore the final energy gain as seen in Fig. 3c (blue curve), is entirely due to the net gain in longitudinal momentum. This is in agreement with the understanding developed using Eq. (11), according to which, in the radiation reaction dominated regime, a monotonic decrease in the parameter $\Delta$ implies energy gain along with increase in forward longitudinal momentum. The energy gain is found to be $\gamma \sim 1.4 \times 10^{4}$ ($\sim 7 \,\, {\rm GeV}$) which is two orders of magnitude higher than the earlier case. The trajectory of the particle is shown in Fig. 4a (blue curve), which clearly shows that in the presence of radiation reaction the particle passes through the focal point. Further the trajectory of the particle clearly exhibits the Doppler shift in the frequency of the wave seen by the particle. Since the parameter $\Delta$ is related to the Doppler shifted frequency as $\omega ^{'} = \Delta \omega _{0}$, the rise and fall of $\Delta$ (blue curve in Fig. 3d) is reflected in the increase and decrease in oscillation frequency observed in the particle trajectory (blue curve in Fig. 4a). We also note, that the dynamics of the charged particle in the presence of radiation reaction as governed by Landau-Lifshitz equation (represented by the magenta curve) shows an excellent match with that obtained using Hartemann-Luhmann equation, implying that the effect of Schott term in the radiation reaction dominated regime is indeed negligible.

We would like to emphasize here that the passage of the particle through the focal point happens only if in the region close to the reflection point radiation reaction term dominates over the Lorentz force term i.e. $\tau _0 a^3_{z_{ref}} \geqslant 1$. For the parameters corresponding to Fig. 3$\tau _0 a^3_{z_{ref}} \sim 12>> 1$. There exists other initial conditions for which, although inequality Eq. (16) is satisfied, the charged particle may still reflect even in the presence of radiation reaction effects. This happens if the initial conditions are such that near the reflection point, the Lorentz force is dominant over the radiation reaction force or in other words the particle reflects before reaching the radiation reaction dominant regime. We now turn to the implications of the second set of initial conditions. In this case, the laser parameters and the initial particle momentum are chosen to be same as in Fig. 3 of the manuscript ($a_0 = 1000$, $\vec{p} = 0$ and $P_{\perp 0} = 0$), but the particle is kept further away from the focal point ($z_0 = -\,9869$); this choice satisfies the inequality Eq. (16) ($a_{z_{ref}} \sim 62.7 < a_0$). The numerical results are presented in Figs. 5 and 6. The Fig. 5a–d respectively represent the longitudinal momentum, transverse momentum, energy and $\Delta$ with respect to “z” co-ordinate and Fig. 6 represents the trajectory of the particle. In all the figures the blue and magenta curves respectively represent the forward and reflected motion of the particle in the absence of radiation reaction effects, whereas red and green curves respectively represent the forward and reflected motion of the charged particle in the presence of radiation reaction effects. It is clearly seen from these figures that the particle reflects from the focal region even in the presence of radiation reaction effects (radiation reaction just pushes the particle a little closer to the focal point). This is because, for these initial conditions, near the reflection point, Lorentz force dominates over the radiation reaction force, $\tau _0 a^3_{z_{ref}} \sim 4 \times 10^{-3} \ll 1$. Comparing Fig. 5 with Fig. 3, we see that in the focused regime dominance of Lorentz force over radiation reaction force or vice-versa is clearly seen in the evolution of the parameter $\Delta$. In Fig. 3, without radiation reaction force, the parameter $\Delta$ monotonically increases in the focused regime in conformity with Eq. (15) of the manuscript, whereas inclusion of radiation reaction force causes the parameter $\Delta$ to monotonically decrease in the focused regime after the reflection point, clearly indicating the dominance of radiation reaction force over the Lorentz force (see Eq. 11). In Fig. 5, for both with and without radiation reaction force, $\Delta$ monotonically increases in the focused regime, which indicates that with these initial conditions Lorentz force always dominates over radiation reaction force. To summarize, if the initial conditions are such that particle is driven into regimes where the radiation reaction force dominates over Lorentz force i.e. $\tau _0 a_0^3 \geqslant 1$, radiation reaction causes the particle to cross the focal region.

For the third set of initial conditions, the particle dynamics is shown in Figs. 7 and 8. In Fig. 7a–d respectively represent the longitudinal and transverse component of particle momentum, the energy and the parameter $\Delta$; and Fig. 8 represents the trajectory of the particle. As before, we first discuss the particle dynamics in the absence of radiation reaction effects. The red curves in Fig. 7a and b respectively show that the average longitudinal momentum decreases (values correspond to y-axis on the left) whereas the amplitude of the transverse momentum increases, as the particle approaches the focal point. The peak value of transverse momentum at the focal point matches $a_0$ ($\mid \vec{p_{\perp }} \mid = a_0 = 1000$), in agreement with Eq. (9). As expected, in this case, the particle passes through the focal point, and the trend of average longitudinal momentum and amplitude of transverse momentum is reversed as it moves away from the focal point. The particle gains average longitudinal momentum and loses transverse momentum in the defocused region. These observations are in agreement with Eqs. (12) and (9). The corresponding energy gain is shown in Fig. 7c (red curve with values corresponding to y-axis on the left), which shows that for the second set of initial conditions, the average energy gain is around $\gamma \sim 700$ ($\sim 0.35 \,\, {\rm GeV}$). Again we note that the average energy gain does not change throughout the motion ($\overline{d \gamma /d \tau } \approx 0$), which is in agreement with Eq. (6). Finally Fig. 7d, shows the evolution of the parameter $\Delta$ as a function of particle position *z* (red curve with values corresponding to y-axis on the left). It monotonically increases upto the focal point and then monotonically decreases in the defocused region. The green curve on the top of the red curve is the plot of analytical expression of $\Delta$ as given by Eq. (15), which clearly shows an excellent agreement with the numerical result. The red curve in Fig. 8, which represents the trajectory in the absence of radiation reaction effects (values correspond to y-axis on the left) shows, as expected, passage through the focal point.

With the third set of initial conditions, the particle dynamics in the presence of radiation reaction effects, is represented by the blue curve in Figs. 7 and 8 with values corresponding to y-axis on the right in Figs. 7a–d and 8. It is found that the particle dynamics is qualitatively similar to that obtained using the first set of initial conditions. The energy gain, as with the first set of initial conditions, is found to be two orders of magnitude larger than that obtained without radiation reaction effects. Thus, as mentioned in the introduction, irrespective of the choice of initial conditions, radiation reaction forces pushes the particle through the high intensity focal point, provided radiation reaction force dominates over Lorentz force and the particle gains energy due to gain in forward longitudinal momentum. In this case also, the particle dynamics as governed by Landau-Lifshitz equation (represented by magenta curve) shows an excellent match with that obtained using Hartemann-Luhmann equation, again indicating that the effect of Schott term in the radiation reaction dominated regime is negligible.

## Summary and conclusions

We have studied the dynamics of a charged particle in a focused light wave by taking account of radiation reaction effects. Basically the dynamics is governed by two effects viz. ponderomotive effects due to focussing and radiation reaction forces. The dynamics have been studied using two well known equations viz. the Hartemann-Luhmann equation and the Landau-Lifshitz equation. We firstly show that both the equations give identical results and secondly, irrespective of the choice of initial conditions, in the presence of radiation reaction, the particle does not reflect from the focal region provided radiation reaction force dominates over Lorentz force, thereby gaining a large amount of energy and forward momentum from the focused light wave.

The fact that both these equations give identical results, although $u^{\alpha }u_{\alpha } = 1$ is not conserved by Hartemann-Luhmann equation, is due to the fact that deviation of $u^{\alpha }u_{\alpha }$ from unity is only due to the absence of Schott term in the Hartemann-Luhmann equation, which in our case is much smaller ($\sim 10^{-8}$ times smaller) than the radiation term. Therefore quantities like energy ($\gamma$). momentum ($\vec{p}$) etc. calculated using Hartemann-Luhmann equation (where Schott term is absent) closely matches with that calculated using Landau-Lifshitz equation of motion (where Schott term is present). For our set of prameters, both $\gamma ^2$ and $p^{2}$ are of order $\sim 10^8$. Thus $\gamma ^2 \sim 0 + {\mathcal {O}}(10^{8}) \sim 10^8$ for Hartemann-Luhmann equation and $\gamma ^2 \sim 1 + {\mathcal {O}}(10^{8}) \sim 10^8$ for Landau-Lifshitz equation. The numerical difference in $u^{\alpha }u_{\alpha } = \gamma ^2 - p^2$ calculated using Hartemann-Luhmann equation and Landau-Lifshitz equation results from cancellation of large numbers. There exists several references in current literature^24,54–56^ where only the radiation term (Schott term neglected) has been used to study the effect of radiation reaction forces. In all such cases, Schott term has been dropped as it has been shown to be much weaker than the radiation term.

Further the result that particle does not reflect from the focal region provided radiation reaction force dominates over Lorentz force is in sharp contrast to the well known result by Kaw et al.^9^, derived in the absence of radiation reaction effects, where for certain initial conditions, the particle reflects from the high intensity region, thereby losing forward energy. Specifically, starting with a co-propagating particle placed initially in the focused region, our numerical studies show that in the absence of radiation reaction, the particle may either reflect (due to ponderomotive deceleration) or may pass through the focal point, respectively depending upon whether it satisfies or violates the inequality given by Eq. (16). In the presence of radiation reaction (when the radiation reaction dominates over Lorentz force), in both the above cases it is found that radiation reaction effects forces the particle to go through the focal point and thereby imparting significant amount of energy. We note here that there are other works for ex. by Gonoskov et al.^57^ and Ji et al.^58^ where it is shown that in the radiation reaction dominated regime, charged particles overcome the ponderomotive force and push their way into regions of high intensity. This is similar to our observation (see Fig. 3), where in the absence of radiation reaction particle reflects before reaching the focal point, whereas in the presence of radiation reaction it passes through the focal point. Through our studies we have clearly differentiated the ponderomotive and radiation reaction effects on a charged particle interacting with a focused light wave. Although our work has been carried using a simplified focusing model, in the above sense, our work supplements the work reported by others. From the perspective of energy gain, our studies clearly show that the parameter space for forward energy gain which is reduced by ponderomotive effects is compensated by radiation reaction effects. The energy gain observed in the presence of radiation reaction, for our set of parameters, is found be two orders of magnitude greater than that obtained without radiation reaction effects; and also this result is independent of the choice of model equation. We have also found that these results are qualitatively the same when studied with a light pulse having a Gaussian envelope. Details of our study on energy gain for a particle interacting with a Gaussian-beam pulse including radiation reaction effects will be reported in a future publication.

## Data Availability

The datasets used and/or analysed during the current study available from the corresponding author on reasonable request.

## References

[1] Landau, L. D. & Lifshitz, E. M. *The Classical Theory of Fields* (Butterworth-Heinemann, 1980).

[2] Gibbon P (2005). Short Pulse Laser Interaction with Matter.

[3] Shebalin JV (1988). An exact solution to the relativistic equation of motion of a charged particle driven by a linearly polarized electromagnetic wave. IEEE Trans. Plasma Sci..

[4] Sagar V, Sengupta S, Kaw P (2012). Exact analysis of particle dynamics in combined field of finite duration laser pulse and static axial magnetic field. Phys. Plasmas.

[5] Kolomenskii A, Lebedev A (1962). The autoresonance motion of a particle in a plane electromagnetic wave. Doklady Akad. Nauk SSSR.

[6] Kolomenskii A, Lebedev A (1963). Self-resonant particle motion in a plane electromagnetic wave. In Soviet Physics Doklady.

[7] Sagar V, Sengupta S, Kaw P (2012). Exact analysis of particle dynamics in combined field of finite duration laser pulse and static axial magnetic field. Phys. Plasmas.

[8] Feldman MJ, Chiao RY (1971). Single-cycle electron acceleration in focused laser fields. Phys. Rev. A.

[9] Kaw PK, Kulsrud RM (1973). Relativistic acceleration of charged particles by superintense laser beams. Phys. Fluids.

[10] Sagar V, Sengupta S, Kaw P (2013). Adiabatic formulation of charged particle dynamics in an inhomogeneous electro-magnetic field. Laser Part. Beams.

[11] Sagar V, Sengupta S, Kaw P (2014). Effect of polarization and focusing on laser pulse driven auto-resonant particle acceleration. Phys. Plasmas.

[12] Wong LJ (2017). Laser-induced linear-field particle acceleration in free space. Sci. Rep..

[13] Li Y (2011). Direct laser acceleration of electron by an ultra intense and short-pulsed laser in under-dense plasma. Phys. Plasmas.

[14] Strickland D, Mourou G (1985). Compression of amplified chirped optical pulses. Opt. Commun..

[15] Perry MD, Mourou G (1994). Terawatt to petawatt subpicosecond lasers. Science.

[16] See http://www.extreme-light-infrastructure.eu/ (2021).

[17] Shen CS (1970). Synchrotron emission at strong radiative damping. Phys. Rev. Lett..

[18] Shen CS (1972). Magnetic bremsstrahlung in an intense magnetic field. Phys. Rev. D.

[19] Hadad Y (2010). Effects of radiation reaction in relativistic laser acceleration. Phys. Rev. D.

[20] Blackburn TG (2020). Radiation reaction in electron-beam interactions with high-intensity lasers. Rev. Modern Plasma Phys..

[21] Mishra Shivam Kumar, Sengupta Sudip (2021). Exact solution of hartemann-luhmann equation of motion for a charged particle interacting with an intense electromagnetic wave/pulse. Eur. Phys. J. Spec. Top..

[22] Di Piazza A, Hatsagortsyan KZ, Keitel CH (2009). Strong signatures of radiation reaction below the radiation-dominated regime. Phys. Rev. Lett..

[23] Piazza A (2008). Radiation reaction in classical electrodynamics. Lett. Math. Phys..

[24] Gong Z, Mackenroth F, Yan X, Arefiev A (2019). Radiation reaction as an energy enhancement mechanism for laser-irradiated electrons in a strong plasma magnetic field. Sci. Rep..

[25] Sagar V, Sengupta S, Kaw PK (2015). Radiation reaction effect on laser driven auto-resonant particle acceleration. Phys. Plasmas.

[26] Russman F, Almansa I, Peter E, Marini S, Rizzato FB (2020). Non-resonant acceleration of charged particles driven by the associated effects of the radiation reaction. J. Plasma Phys..

[27] Harvey C, Marklund M (2012). Radiation damping in pulsed gaussian beams. Phys. Rev. A.

[28] Lorentz HA (1909). The Theory of Electrons.

[29] Dirac PAM (1928). Proc. R. Soc. A.

[30] Dirac PAM (1938). Proc. R. Soc. A.

[31] Abraham M (1905). Theorie der Elektrizität.

[32] Griffiths, D. J. *Introduction to Electrodynamics*; 4th ed. (Pearson, 2013). (**Re-published by Cambridge University Press in 2017**).

[33] Jackson JD (1999). Classical Electrodynamics.

[34] Rohrlich, F. *Classical Charged Particles 3rd Edition (World Scientific, 2007* (Syracuse University, 2007).

[35] Rohrlich F (2000). The self-force and radiation reaction. Am. J. Phys..

[36] Kasher JC (1976). One-dimensional central-force problem, including radiation reaction. Phys. Rev. D.

[37] Bhabha, H. J. Magnetism. *Proc. R. Soc. A* **117**, 148 (1939).

[38] Connell RFO (2003). The equation of motion of an electron. Phys. Rev. A.

[39] Ford GW, O’Connell RF (1991). Radiation reaction in electrodynamics and the elimination of runaway solutions. Phys. Rev. A.

[40] Ford GW, O’Connell RF (1993). Relativistic form of radiation reaction. Phys. Rev. A.

[41] Eliezer CJ (1948). On the classical theory of particles. Proc. R. Soc. Lond. A.

[42] Mo T, Papas C (1971). New equation of motion for classical charged particles. Phys. Rev. D.

[43] Caldirola P (1979). A relativistic theory of the classical electron. Riv. Nuovo Cimento Soc. Ital. Fis..

[44] Hartemann FV, Luhmann NC (1995). Classical electrodynamical derivation of the radiation damping force. Phys. Rev. Lett..

[45] Yaremko Y (2013). Exact solution to the landau-lifshitz equation in a constant electromagnetic field. J. Math. Phys..

[46] Sokolov I (2009). Renormalization of the Lorentz-Abraham-Dirac equation for radiation reaction force in classical electrodynamics. J. Exp. Theor. Phys..

[47] Sokolov IV, Naumova NM, Nees JA, Mourou GA, Yanovsky VP (2009). Dynamics of emitting electrons in strong laser fields. Phys. Plasmas.

[48] Sokolov IV, Nees JA, Yanovsky VP, Naumova NM, Mourou GA (2010). Emission and its back-reaction accompanying electron motion in relativistically strong and qed-strong pulsed laser fields. Phys. Rev. E.

[49] Bellotti U, Bornatici M (1997). Energy conservation equation for a radiating pointlike charge in the context of the abraham-lorentz versus the abraham-becker radiation-reaction force. Phys. Rev. E.

[50] Laufer G (1996). Introduction to Optics and Lasers in Engineering.

[51] Bogolyubov, N. & Mitropolskii, Y. A. *Asymptotic methods in the theory of nonlinear oscillations* (Tech. Rep, FOREIGN TECHNOLOGY DIV WRIGHT-PATTERSON AFB OHIO, 1955).

[52] Nicholson D (1983). Introduction to Plasma Theory.

[53] Landau L, Lifshitz E, Sykes J, Bell J (1976). Mechanics: Volume 1 Course of theoretical physics.

[54] Esirkepov, T. Z. *et al*. Attractors and chaos of electron dynamics in electromagnetic standing wave. arXiv:1412.6028 (2014).

[55] Yuan Y, Nalewajko K, Zrake J, East WE, Blandford RD (2016). Kinetic study of radiation-reaction-limited particle acceleration during the relaxation of unstable force-free equilibria. Astrophys. J..

[56] Zhang Y, Krasheninnikov S (2019). Radiation friction force effects on electron dynamics in ultra-intensity laser pulse. Phys. Plasmas.

[57] Gonoskov A (2014). Anomalous radiative trapping in laser fields of extreme intensity. Phys. Rev. Lett..

[58] Ji LL, Pukhov A, Kostyukov IY, Shen BF, Akli K (2014). Radiation-reaction trapping of electrons in extreme laser fields. Phys. Rev. Lett..

